# Supplementary material for: Pyruvate Dehydrogenase Kinase 1 inhibition mediated oxidative phosphorylation enhancement in cartilage promotes osteoarthritis progression
Source: BMC Musculoskelet Disord. 2023 Jul 20;24:597. doi: 10.1186/s12891-023-06585-6 (PMC10357736; doi:10.1186/s12891-023-06585-6)
Supplement: Supplementary file 1 — Additional file 1. [file 12891_2023_6585_MOESM1_ESM.zip › MF.Gsea.1653627316242/GOMF_HYDRO_LYASE_ACTIVITY.html]

Details for gene set GOMF\_HYDRO\_LYASE\_ACTIVITY[GSEA]

|  || Dataset | 55457\_collapsed\_to\_symbols.cls.cls #OA\_versus\_normal.cls.cls #OA\_versus\_normal\_repos |
| Phenotype | cls.cls#OA\_versus\_normal\_repos |
| Upregulated in class | OA |
| GeneSet | GOMF\_HYDRO\_LYASE\_ACTIVITY |
| Enrichment Score (ES) | 0.3048886 |
| Normalized Enrichment Score (NES) | 1.2364345 |
| Nominal p-value | 0.15223098 |
| FDR q-value | 0.4741186 |
| FWER p-Value | 1.0 |
Table: GSEA Results Summary

  

Fig 1: Enrichment plot: GOMF\_HYDRO\_LYASE\_ACTIVITY      
 Profile of the Running ES Score & Positions of GeneSet Members on the Rank Ordered List

  

| SYMBOL | TITLE | RANK IN GENE LIST | RANK METRIC SCORE | RUNNING ES | CORE ENRICHMENT || 1 | ACO2 | aconitase 2 [Source:HGNC Symbol;Acc:HGNC:118] | 290 | 0.756 | 0.0320 | Yes |
| 2 | UROS | uroporphyrinogen III synthase [Source:HGNC Symbol;Acc:HGNC:12592] | 323 | 0.735 | 0.0826 | Yes |
| 3 | APIP | APAF1 interacting protein [Source:HGNC Symbol;Acc:HGNC:17581] | 643 | 0.607 | 0.1016 | Yes |
| 4 | HADHB | hydroxyacyl-CoA dehydrogenase trifunctional multienzyme complex subunit beta [Source:HGNC Symbol;Acc:HGNC:4803] | 675 | 0.599 | 0.1424 | Yes |
| 5 | NAXD | NAD(P)HX dehydratase [Source:HGNC Symbol;Acc:HGNC:25576] | 886 | 0.547 | 0.1655 | Yes |
| 6 | FAHD2A | fumarylacetoacetate hydrolase domain containing 2A [Source:HGNC Symbol;Acc:HGNC:24252] | 958 | 0.527 | 0.1979 | Yes |
| 7 | HSD17B4 | hydroxysteroid 17-beta dehydrogenase 4 [Source:HGNC Symbol;Acc:HGNC:5213] | 1354 | 0.450 | 0.1996 | Yes |
| 8 | PARK7 | Parkinsonism associated deglycase [Source:HGNC Symbol;Acc:HGNC:16369] | 1406 | 0.442 | 0.2276 | Yes |
| 9 | ECI1 | enoyl-CoA delta isomerase 1 [Source:HGNC Symbol;Acc:HGNC:2703] | 1453 | 0.435 | 0.2554 | Yes |
| 10 | TGDS | "TDP-glucose 4,6-dehydratase [Source:HGNC Symbol;Acc:HGNC:20324]" | 1628 | 0.409 | 0.2714 | Yes |
| 11 | DGLUCY | D-glutamate cyclase [Source:HGNC Symbol;Acc:HGNC:20498] | 1768 | 0.388 | 0.2886 | Yes |
| 12 | ECHS1 | "enoyl-CoA hydratase, short chain 1 [Source:HGNC Symbol;Acc:HGNC:3151]" | 1925 | 0.369 | 0.3031 | Yes |
| 13 | IREB2 | iron responsive element binding protein 2 [Source:HGNC Symbol;Acc:HGNC:6115] | 2215 | 0.337 | 0.3049 | Yes |
| 14 | PTGIS | prostaglandin I2 synthase [Source:HGNC Symbol;Acc:HGNC:9603] | 2654 | 0.284 | 0.2913 | No |
| 15 | ENO3 | enolase 3 [Source:HGNC Symbol;Acc:HGNC:3354] | 3207 | 0.230 | 0.2649 | No |
| 16 | PCBD1 | pterin-4 alpha-carbinolamine dehydratase 1 [Source:HGNC Symbol;Acc:HGNC:8646] | 3405 | 0.211 | 0.2648 | No |
| 17 | ALAD | aminolevulinate dehydratase [Source:HGNC Symbol;Acc:HGNC:395] | 3513 | 0.201 | 0.2710 | No |
| 18 | CA3 | carbonic anhydrase 3 [Source:HGNC Symbol;Acc:HGNC:1374] | 3671 | 0.188 | 0.2723 | No |
| 19 | GMDS | "GDP-mannose 4,6-dehydratase [Source:HGNC Symbol;Acc:HGNC:4369]" | 4151 | 0.142 | 0.2453 | No |
| 20 | HADHA | hydroxyacyl-CoA dehydrogenase trifunctional multienzyme complex subunit alpha [Source:HGNC Symbol;Acc:HGNC:4801] | 4163 | 0.141 | 0.2546 | No |
| 21 | CA5B | carbonic anhydrase 5B [Source:HGNC Symbol;Acc:HGNC:1378] | 4333 | 0.125 | 0.2505 | No |
| 22 | TBXAS1 | thromboxane A synthase 1 [Source:HGNC Symbol;Acc:HGNC:11609] | 4338 | 0.125 | 0.2592 | No |
| 23 | CA14 | carbonic anhydrase 14 [Source:HGNC Symbol;Acc:HGNC:1372] | 4753 | 0.091 | 0.2336 | No |
| 24 | CDYL | chromodomain Y like [Source:HGNC Symbol;Acc:HGNC:1811] | 5016 | 0.072 | 0.2183 | No |
| 25 | CBS | cystathionine beta-synthase [Source:HGNC Symbol;Acc:HGNC:1550] | 5285 | 0.049 | 0.2010 | No |
| 26 | HACD1 | 3-hydroxyacyl-CoA dehydratase 1 [Source:HGNC Symbol;Acc:HGNC:9639] | 5342 | 0.044 | 0.1998 | No |
| 27 | ENO2 | enolase 2 [Source:HGNC Symbol;Acc:HGNC:3353] | 5572 | 0.026 | 0.1839 | No |
| 28 | FH | fumarate hydratase [Source:HGNC Symbol;Acc:HGNC:3700] | 5729 | 0.013 | 0.1727 | No |
| 29 | HACD3 | 3-hydroxyacyl-CoA dehydratase 3 [Source:HGNC Symbol;Acc:HGNC:24175] | 5755 | 0.011 | 0.1716 | No |
| 30 | AUH | AU RNA binding methylglutaconyl-CoA hydratase [Source:HGNC Symbol;Acc:HGNC:890] | 5911 | 0.001 | 0.1596 | No |
| 31 | CYP1B1 | cytochrome P450 family 1 subfamily B member 1 [Source:HGNC Symbol;Acc:HGNC:2597] | 5925 | 0.000 | 0.1587 | No |
| 32 | CA9 | carbonic anhydrase 9 [Source:HGNC Symbol;Acc:HGNC:1383] | 6142 | -0.015 | 0.1430 | No |
| 33 | HACD2 | 3-hydroxyacyl-CoA dehydratase 2 [Source:HGNC Symbol;Acc:HGNC:9640] | 6162 | -0.017 | 0.1427 | No |
| 34 | CA10 | carbonic anhydrase 10 [Source:HGNC Symbol;Acc:HGNC:1369] | 6360 | -0.032 | 0.1297 | No |
| 35 | CA8 | carbonic anhydrase 8 [Source:HGNC Symbol;Acc:HGNC:1382] | 6397 | -0.035 | 0.1294 | No |
| 36 | CA11 | carbonic anhydrase 11 [Source:HGNC Symbol;Acc:HGNC:1370] | 6895 | -0.072 | 0.0959 | No |
| 37 | CA7 | carbonic anhydrase 7 [Source:HGNC Symbol;Acc:HGNC:1381] | 7210 | -0.097 | 0.0785 | No |
| 38 | ECHDC3 | enoyl-CoA hydratase domain containing 3 [Source:HGNC Symbol;Acc:HGNC:23489] | 7215 | -0.097 | 0.0851 | No |
| 39 | ALOXE3 | arachidonate lipoxygenase 3 [Source:HGNC Symbol;Acc:HGNC:13743] | 7502 | -0.119 | 0.0714 | No |
| 40 | CA12 | carbonic anhydrase 12 [Source:HGNC Symbol;Acc:HGNC:1371] | 7520 | -0.120 | 0.0788 | No |
| 41 | ECHDC1 | ethylmalonyl-CoA decarboxylase 1 [Source:HGNC Symbol;Acc:HGNC:21489] | 7532 | -0.121 | 0.0867 | No |
| 42 | CA1 | carbonic anhydrase 1 [Source:HGNC Symbol;Acc:HGNC:1368] | 7585 | -0.126 | 0.0917 | No |
| 43 | CA2 | carbonic anhydrase 2 [Source:HGNC Symbol;Acc:HGNC:1373] | 7708 | -0.135 | 0.0919 | No |
| 44 | EHHADH | enoyl-CoA hydratase and 3-hydroxyacyl CoA dehydrogenase [Source:HGNC Symbol;Acc:HGNC:3247] | 7968 | -0.153 | 0.0828 | No |
| 45 | CYP1A1 | cytochrome P450 family 1 subfamily A member 1 [Source:HGNC Symbol;Acc:HGNC:2595] | 8195 | -0.171 | 0.0775 | No |
| 46 | CA6 | carbonic anhydrase 6 [Source:HGNC Symbol;Acc:HGNC:1380] | 9260 | -0.253 | 0.0129 | No |
| 47 | ACO1 | aconitase 1 [Source:HGNC Symbol;Acc:HGNC:117] | 9426 | -0.266 | 0.0193 | No |
| 48 | ENO1 | enolase 1 [Source:HGNC Symbol;Acc:HGNC:3350] | 9606 | -0.284 | 0.0258 | No |
| 49 | CA4 | carbonic anhydrase 4 [Source:HGNC Symbol;Acc:HGNC:1375] | 10464 | -0.369 | -0.0142 | No |
| 50 | ECHDC2 | enoyl-CoA hydratase domain containing 2 [Source:HGNC Symbol;Acc:HGNC:23408] | 11406 | -0.488 | -0.0523 | No |
| 51 | ENOSF1 | enolase superfamily member 1 [Source:HGNC Symbol;Acc:HGNC:30365] | 11640 | -0.524 | -0.0327 | No |
| 52 | CYP1A2 | cytochrome P450 family 1 subfamily A member 2 [Source:HGNC Symbol;Acc:HGNC:2596] | 11719 | -0.536 | -0.0001 | No |
| 53 | CA5A | carbonic anhydrase 5A [Source:HGNC Symbol;Acc:HGNC:1377] | 12145 | -0.630 | 0.0123 | No |
| 54 | FASN | fatty acid synthase [Source:HGNC Symbol;Acc:HGNC:3594] | 12194 | -0.643 | 0.0550 | No |
Table: GSEA details [plain text format]

  

Fig 2: GOMF\_HYDRO\_LYASE\_ACTIVITY      
 Blue-Pink O' Gram in the Space of the Analyzed GeneSet

  

Fig 3: GOMF\_HYDRO\_LYASE\_ACTIVITY: Random ES distribution      
 Gene set null distribution of ES for **GOMF\_HYDRO\_LYASE\_ACTIVITY**
